# Conceptual biology, hypothesis discovery, and text mining: Swanson's legacy

**DOI:** 10.1186/1742-5581-3-2

**Published:** 2006-04-03

**Authors:** Tanja Bekhuis

**Affiliations:** 1 Department of Library & Information Science, School of Information Sciences, University of Pittsburgh, 135 North Bellefield Avenue, Pittsburgh, PA 15260, USA. Current address: Department of Biology, Juniata College, 1700 Moore Street, Huntingdon, PA 16652, USA

## Abstract

Innovative biomedical librarians and information specialists who want to expand their roles as expert searchers need to know about profound changes in biology and parallel trends in text mining. In recent years, conceptual biology has emerged as a complement to empirical biology. This is partly in response to the availability of massive digital resources such as the network of databases for molecular biologists at the National Center for Biotechnology Information. Developments in text mining and hypothesis discovery systems based on the early work of Swanson, a mathematician and information scientist, are coincident with the emergence of conceptual biology. Very little has been written to introduce biomedical digital librarians to these new trends. In this paper, background for data and text mining, as well as for knowledge discovery in databases (KDD) and in text (KDT) is presented, then a brief review of Swanson's ideas, followed by a discussion of recent approaches to hypothesis discovery and testing. 'Testing' in the context of text mining involves partially automated methods for finding evidence in the literature to support hypothetical relationships. Concluding remarks follow regarding (a) the limits of current strategies for evaluation of hypothesis discovery systems and (b) the role of literature-based discovery in concert with empirical research. Report of an informatics-driven literature review for biomarkers of systemic lupus erythematosus is mentioned. Swanson's vision of the hidden value in the literature of science and, by extension, in biomedical digital databases, is still remarkably generative for information scientists, biologists, and physicians.

## Introduction

When biomedical researchers pose reference questions in the context of conceptual biology, librarians and information specialists may be puzzled. Their patrons probably want to generate and test hypotheses, often molecular ones, based on information located in biological and bibliometric databases. Innovative information professionals with requisite skills and motivation can add value to the usual array of services by expanding their roles as expert searchers. To start, they need to know about profound changes in biology and parallel trends in text mining – a kind of computerized data mining to search for meaningful patterns of text, such as strings of nucleotides or clinical concepts in databases annotated by expert humans.

## The emergence of conceptual biology and text mining

Biologists view testable and falsifiable scientific hypotheses as superior to theoretical models because they value empirical evidence. In fact, the phrase 'theoretical biology' is considered by some to be a contradiction in terms [1]. Nevertheless, the abundance of digital information, especially in molecular and cellular biology, is such a promising resource that conceptual – theoretical and not empirical – literature-based approaches for generating and testing hypotheses are emerging. Cognizant of this trend, Blagosklonny and Pardee argue in an essay published in *Nature *[2] that conceptual biology is an important complement to empirical biology in part because conceptual reviews of enormous databases overcome the obstacles of their "complexity and overproduction" (p. 373). In other words, digital databases represent an opportunity for scientific exploration because "retrievable facts are being accumulated in databases, from a variety of sources in seemingly unrelated fields, and from thousands of journals" (p. 373). Although the pioneer of bibliographic knowledge discovery is neither mentioned by Bray nor Blagosklonny and Pardee, their comments are reminiscent of Swanson's. Consider the following quote:

The reward system and ethos of science ... recognize only the physical world as a source of new knowledge. The literature tends to be seen as a sort of knowledge necrology, a mechanism of diffusion that supports laboratory-based discovery, but without a life of its own. Science may be better served by a new image of its literature as a vast mosaic of undiscovered connections, a potential source of countless recombinant ideas – a world with its own endless frontier (p. 36) [3].

Today, biologists are beginning to embrace Swanson's prescient notions, as evidenced by Bray [1], Blagosklonny and Pardee [2], and the appearance of journals such as *Theoretical Biology and Medical Modelling *[4]. Moreover, developers of text or literature mining applications are working at a furious pace, in part because mapping the human genome led to an explosion of text-based genetic information. As a result, several large and complicated genomics and proteomics databases exist. (Genomics refers to the study of an organism's genome or full complement of genetic information. Proteomics refers to the study of an organism's proteome or full complement of proteins encoded by its genome.) Many specialized, overlapping databases exist for biomedical researchers and molecular biologists interested in studying structure, function, and interactions among genes and proteins. For example, see the National Center for Biotechnology Information's catalog of resources [5]. These digital databases are information rich, but still relatively opaque without mining tools.

Powerful trends are in place for continued development of text mining (TM) applications useful for generating hypotheses and for finding evidence to support hypotheses. First, TM tools facilitate conceptually driven, more efficient retrieval – an advance that scholars exposed to a superabundance of information will welcome [6]. Second, TM tools can bridge disjoint literatures unknown to researchers who have specialized in response to information overload [7]. Third, the typical topography of information networks is characterized by directed clusters of nodes such that searching in one "continent" might preclude access to another [8]. Hence, TM tools can help bridge information continents on the Web and other scale-free networks. Fourth, TM tools can stem the profligate waste of digital library resources by enhancing access and adding value to content.

Aside from a few review papers [9-12] and in the introductions to papers describing particular TM methods or tools [13-15], very little has been written to introduce digital librarians to TM and hypothesis discovery. Hence, in this paper, background for mining and knowledge discovery is presented, then a brief review of Swanson's ideas, followed by a discussion of recent approaches to hypothesis discovery (generation) and testing. 'Testing hypotheses' in the context of literature-based TM refers to partially automated processes for finding evidence to support hypothetical relationships. A major goal of informaticians working in concert with subject experts is to unearth enough evidence in support of hypotheses that will be of interest to empiricists for eventual experimental validation.

## Mining and knowledge discovery

Data mining refers to the automated search for meaningful patterns of data (including text) stored in very large digital databases or distributed over the Web. The term 'data mining' was popularized in the 1990s when corporations developed data warehouses to store the deluge of digital information. Early resources for mining were structured relational databases of numeric data. Today, data types may be numeric, textual, visual, and more. If textual, data may be unstructured, such as full text documents, or partially structured, such as MEDLINE abstracts, tagged HTML documents, or annotated databases. However, some see full text as inherently semi-structured because of grammatical rules for natural language and conventions for document structure [10]. Structure has methodological implications for text mining. For example, consider the several sections of a scientific article: title, abstract, keyword list, introduction, methods, results, discussion, and reference list. Since these vary with respect to type and amount of information, extraction of information can be "tuned" to the section [16].

Many different TM methods exist [17], including some that use co-citations, author names, journal names, words, phrases, emails, technical support transcripts, patient records, and gene or protein sequences. Even though TM methods need not be used for theoretical model building or testing, Srinivasan believes that "text mining applied to the domain of biomedicine is conceptual biology" (p. 410) [18]. This may be an overstatement. Nevertheless, text mining in tandem with conceptual biology is a potentially powerful strategy for finding novel relationships in literature-based databases, such as MEDLINE.

Regardless of purpose, successful mining adds value to retrieved information by imposing a meaningful structure on what could otherwise be an incomprehensible morass. Methods vary with the disciplinary focus of developers and include statistical, linguistic, and visual approaches. Additionally, mining data may be thought of as a step in the cycle of knowledge discovery in databases (KDD) or as intrinsic to the entire cycle [19,20]. In either case, a primary goal of KDD is to map low-level data into more meaningful forms. The iterative cycle of KDD can involve problem definition, information retrieval, data cleaning, statistical or linguistic information extraction, analysis, visual display, and interpretation. Developers have tried to fully automate the cycle, but human experts still need to evaluate results – both interim and final – making decisions at various strategic points throughout the cycle.

More recently, with the rapid development of methods to automate retrieval, extraction, and mining of rich text-based resources in biology, a new term has emerged – knowledge discovery in text (KDT) [10]. Natarajan et al. define KDT as "the process of identifying and extracting valid, *novel *[italics added], potentially useful and ultimately understandable patterns in natural-language documents" (p. 32). The three main phases of KDT, in their view, include (a) information retrieval of relevant documents; (b) information extraction of entities (e.g., gene or protein names), relations (e.g., protein-protein interactions), or events (e.g., molecular pathways); and (c) text mining to find "non-trivial, implicit, previously unknown" patterns (p. 33). Two basic TM tasks are classification and clustering of retrieved documents.

## Swanson's ideas

'Undiscovered public knowledge' is a phrase coined by Swanson [21]. It refers to published knowledge effectively buried in disjoint topical domains –'disjoint' because researchers working in disparate fields are unaware of one another. Hence, truly disjoint literatures have no articles in common. Swanson suggested in a series of creative papers that novel information might be unearthed by systematically studying seemingly unrelated and non-interactive research literatures, which he called "complementary but disjoint" (p. 280) [22]. To demonstrate the feasibility of his ideas, he found evidence for previously overlooked relationships between fish oil and Raynaud's syndrome [23], magnesium and migraine [24], somatomedin C and arginine [25], and viruses as weapons [26]. This is quite remarkable given that Swanson is a mathematician and an information scientist, not a physician.

For readers interested in the methods of Swanson and colleagues, a good place to begin is with Swanson and Smalheiser [27]. A concise summary of an early model described in their paper is offered here: Given a particular research question in biomedicine, a primary goal is to identify two complementary but disjoint literatures *AB *and *BC*, where *A, B*, and *C *are variables or concepts of interest. Begin by searching titles in MEDLINE relevant to *C *and then *A*; review the results and generate a list of titles by shared terms *B*. Taken together, *AB *and *BC *are disjoint since nothing has been published linking *A *with *C*. For example, let *C *represent the source literature on migraine; *A *the target literature on magnesium; and *B *the intermediate literature linking *A *to *C*. After expert review, the shared *B *list of terms in titles of *AB *and *BC *ultimately suggest several testable and novel hypotheses regarding the physiological effects of magnesium deficiency with respect to migraine. At this point, even though a set of hypothesized relationships has been discovered, independent experimental tests are still necessary to validate the results, e.g., by conducting clinical trials.

To partially automate their method, Swanson and Smalheiser developed an interactive software program called ARROWSMITH available on the Internet at two sites [28,29]. The two versions vary somewhat algorithmically and potential users should review both sites before selecting one over the other. Additionally, the latter site seems more 'user friendly' but parts of it are under construction (as of March 2006). At the first site, the user selects one of two modes (hypothesis generation or hypothesis testing) to produce an *A *list and a *C *list of terms by searching MEDLINE titles and medical subject headings via PubMed or OVID. (In the early literature, the two modes are referred to as procedures I and II, respectively, depending on whether or not the user hypothesizes a relationship between *A *and *C *at the outset. Today, the first procedure is sometimes characterized as open and the second as closed.) The hypothesis-testing mode relaxes the early assumption of purely disjoint literature pairs since if one knows of a possible relationship, articles mentioning *A, B*, and *C *probably exist but are not commonly known.

Swanson and Smalheiser [27] recognized that two literatures might be spuriously linked because of shared language in the larger discipline, e.g., medicine. They described several filters in the early version of ARROWSMITH that (a) control this potential confound and (b) introduce human intelligence into the interactive system. The early filters included an a priori stop list of several thousand words (human not machine made), a statistical cutoff for retaining terms based on relative frequency, and category restrictions, e.g., 'dietary factor' or 'toxin.' The recent version of ARROWSMITH offers additional filters, such as 'first publication date.'

Since KDT embraces many different types of studies, it is helpful to have a name for the class of studies deriving from Swanson's earliest insights. Stegmann and Grohmann [30] proposed the term 'Swanson Linking' (SL) for "literature-based discovery where SL may be defined as finding disjoint literature partners by establishing meaningful links between them using information retrieval from bibliographic databases" (p. 112). Following Stegmann and Grohmann, the projects described below could be classified as SL studies. However, the definition may need to be broadened in the future to include all types of databases.

## Swanson linking studies and development

Researchers who extend Swanson's ideas remain faithful to his logic, but are perhaps too respectful of his methods. For example, hypotheses in SL studies usually involve a disease; the database of choice is usually MEDLINE; and evaluation almost always entails replicating Swanson's earliest findings – a strategy probably first adopted by Gordon and Lindsay [31] and Swanson and Smalheiser [27]. Even so, researchers have made major contributions by systematizing Swanson's early methods, improving automation of certain aspects of hypothesis discovery, and mining entities other than titles. A list of chronologically ordered papers from 1986 to 2001 on literature-based discovery is available on the Internet [32].

### Gordon and Lindsay

In 1996, Gordon and Lindsay [31] published a study on discovery support systems because "no other investigators [had] reported conducting literature-based discovery experiments that confirm, disconfirm, or extend Swanson's work in any way" (p. 117). This was a decade after publication of Swanson's first text mining papers. Their results gave credence to Swanson's strategy by confirming the link between Raynaud's syndrome and dietary fish oil. Moreover, they introduced lexical and statistical methods for mining abstracts instead of titles and developed computer-based tools to support discovery. By comparing several frequency measures for choosing terms, they introduced quantitative rigor to the field.

### Weeber *et al.*

Weeber and colleagues [33] developed a concept-based, Natural Language Processing system called DAD (Drug-Adverse Drug Reaction-Disease) to assist biomedical experts in formulating and testing hypotheses, primarily for drug discovery studies. They bypassed the difficulties of extracting words – obviating the need for stop lists and complex queries for synonyms and variants – by mapping words in titles and abstracts to concepts in the Unified Medical Language System (UMLS) Metathesaurus, one of three components in the National Library of Medicine's UMLS [34]. Mapping also facilitates (a) extraction of compound phrases, such as 'blood pressure' and (b) narrowing the search space by using UMLS semantic types as filters. As of March 2006, the UMLS Semantic Network contains 135 semantic types; at least one semantic type is assigned to each of more than one million biomedical concepts. The judicious use of semantic filters, such as 'gene or genome' or 'cell function,' could broaden the kinds of hypotheses generated to date.

To demonstrate the usefulness of their discovery system, Weeber et al. [35] published the results of an interesting study on potentially new target diseases for the drug thalidomide. They found bibliographic evidence in PubMed suggesting that thalidomide could be an effective treatment for chronic hepatitis C, myasthenia gravis, *Helicobacter pylori*-induced gastritis, and acute pancreatitis.

### Stegmann and Grohmann

Stegmann and Grohmann [30] extended SL methodology by employing co-word analysis, a statistical method useful for clustering. Instead of words or concepts, they analyzed strength of co-occurrence for pairs of keywords assigned to MEDLINE documents in the retrieval sets. Keywords included medical subject headings (MeSH), as well as Enzyme Commission Numbers and Chemical Abstracts Service Registry Numbers in the RN field. The analyses lead to maps or "strategical diagrams" of clusters containing keywords. Promising terms linking complementary but disjoint literatures tend to appear in regions of low centrality and density. They validated their approach by replicating Swanson's findings for Raynaud's syndrome and fish oil, and for migraine and magnesium. They also found evidence for a relationship between prions, neurodegenerative diseases, and manganese. This relationship had been mapped earlier by Chen in the context of latent domains of knowledge –'latent' because of the low citation rate of an important paper described in Chen's book on mapping (chapter 7, pp. 216–219) [36].

An advantage of co-word analysis and clustering is that early phases of term selection are automated. However, subject experts still need to review clusters for final selection of appropriate terms. Another strength is that users may find it easier to review maps or diagrams of clusters than long lists of sorted terms. A disadvantage is that the method depends on keywords from a controlled vocabulary. Other methods, such as mining titles and abstracts, are more appropriate if keywords are missing. Additionally, this consideration will be important in the future when researchers try to merge and mine information from databases without shared vocabularies.

### Srinivasan

Srinivasan [18] published the results of an extensive replication of Swanson and Smalheiser's work, carefully comparing her methods to theirs, as well as to those of Gordon and Lindsay, and Weeber et al. She has an active TM program of research and is dedicated to building a "suite of text mining tools that may be used by a domain expert to explore a text collection for hypothesis generation" (p. 397). Additionally, Srinivasan and Libbus published reports of interesting applications that demonstrate the usefulness of her system, such as an SL study exploring the therapeutic benefits of *Curcuma longa *(curcumin) for retinal diseases, Crohn's disease, and spinal cord disorders [37]. Her work resembles Weeber et al. [33] and Stegmann and Grohmann [30] in that she uses UMLS semantic types and MEDLINE metadata (MeSH terms), respectively. However, she combines these elements in a manner very different from either group.

Srinivasan's TM algorithms for discovery entail building profiles of research topics based on weighted MeSH terms from retrieved MEDLINE documents, where weights are estimated within semantic type. Taken together, weighted terms constitute a profile of the topic of interest. For example, a profile for the hereditary disorder Marfans syndrome probably would consist of heavily weighted terms for "genes, proteins, symptoms, drug treatments, other disease, and population groups" (p. 397) [18]. Topics for profiling can be single words or phrases that need not be composed of MeSH terms. Unlike Stegmann and Grohmann [30], the results are ranked term lists rather than clusters.

## Conclusion

Developers commonly try to replicate Swanson's early findings as a means of system appraisal because (a) much of Swanson's work has been validated independently and empirically by clinical researchers and (b) no other agreed-upon criteria exist, with the exception of expert opinion regarding relevancy of results and feasibility of hypotheses. In this context, appraisal implies evaluation of the goodness of sets of discovered hypothetical relationships. If no other criteria for demonstrating validity exist, evaluation must await tests by empiricists who happen to find the results interesting [9]. This is a major problem for developers of hypothesis generating systems.

However, a variant of this approach is possible. Developers could work retrospectively on other well-known, empirically validated phenomena by mining the relevant literature up to meaningful cutoff dates. The goodness of the results sets would depend on whether known causal or temporal relationships are recovered. This is similar to using Swanson's early findings as evaluation criteria, but opens the discovery process to other domains in basic and applied research, such as molecular biology, chemistry, physical therapy, nursing, or public health.

Regardless of disciplinary focus, it is probable that researchers will want to retrieve and merge information from several kinds of databases. This assumes continued interest in interdisciplinary research and expansion of overlapping databases. For example, to glimpse the interconnectedness of databases already available for molecular biologists, visit the National Center for Biotechnology Information website [38] and select one of the nodes of the graphic for Entrez, the integrated system for search and retrieval. This leads to a display of the number of links between databases. These are not symmetric – for example, the number of links between PubMed and Cancer Chromosomes depends on whether one selects PubMed (8,016 links) or Cancer Chromosomes (50,051). This asymmetry will have an impact on future merging and mining efforts.

Currently, hypothesis discovery systems are still in the early phase of development, at least from the perspective of potential users. Nevertheless, in addition to probing appropriate methods for extraction and analysis, it would behoove developers to participate with research teams working on substantive rather than methodological problems. Otherwise, the mainstream biomedical community will ignore results of SL studies, no matter how fascinating.

Additionally, the phrases 'hypothesis testing' and 'knowledge discovery' in the context of text mining are not credible to experimentalists trained in the positivist tradition. Since the appropriate use of text-based, discovery methods is exploratory and therefore useful in early phases of research programs or in proof-of-concept studies, a more general phrase, such as 'exploratory mining' might be more acceptable.

Once the role of discovery methods in research programs is clarified, partnerships with the biomedical community will develop apace. Certainly, the timing is auspicious given greater acceptance of conceptual and computational biology, as well as rapid development of text mining tools. As an example of growing awareness of the potential for discovery methods, consider the following comment by the Director of the Office of Scientific Interchange at the National Institutes of Arthritis and Musculoskeletal and Skin Diseases:

The comprehensive overview of an entire literature with respect to a single question is now in transition. New tools in informatics are making it possible to fuel the search for biomarkers for SLE [systemic lupus erythematosus] ... rapidly and with nuance. Rather than looking for articles using the same key words, or for bibliographic citations in a work of interest, the entire database of medical literature can be probed.... (pp. 223–224) [39]

In support of her suggestion for an informatics-driven review of literature, Mittleman cites several Swanson papers and therefore is aware of the origins of text mining for discovery. It seems clear that Swanson's vision of the hidden value in the literature of science and, by extension, in biomedical digital databases, is still remarkably generative for information scientists, biologists, and physicians. Innovative librarians and information professionals could respond to the changing information needs of their patrons by monitoring developments in KDT, and by acquiring the necessary skills to help patrons locate and mine appropriate databases. Major health sciences libraries could build computational biology centers modeled after Princeton University's Data and Statistical Services (DSS) in the Harvey S. Firestone Memorial Library. Although the DSS unit is not dedicated to biology, the idea of offering consulting services to a particular community is apropos. Even without a dedicated center, one or more librarians could be trained in KDT methods to help biomedical researchers and conceptual biologists locate information useful for generating and testing hypotheses.

## Competing interests

The author(s) state that they have no competing interests.

## Authors' contributions

TB is the sole contributor.
